# Innovating the Concept and Practice of Two-Dimensional Gel Electrophoresis in the Analysis of Proteomes at the Proteoform Level

**DOI:** 10.3390/proteomes7040036

**Published:** 2019-10-30

**Authors:** Xianquan Zhan, Biao Li, Xiaohan Zhan, Hartmut Schlüter, Peter R. Jungblut, Jens R. Coorssen

**Affiliations:** 1 Key Laboratory of Cancer Proteomics of Chinese Ministry of Health, Central South University, 87 Xiangya Road, Changsha 410008, China; 2 Hunan Engineering Laboratory for Structural Biology and Drug Design, Central South University, 87 Xiangya Road, Changsha 410008, China; 3 State Local Joint Engineering Laboratory for Anticancer Drugs, Central South University, 87 Xiangya Road, Changsha 410008, China; 4 National Clinical Research Center for Geriatric Disorders, Central South University, 88 Xiangya Road, Changsha 410008, China; 5 Institute of Clinical Chemistry and Laboratory Medicine, Mass Spectrometric Proteomics, Campus Forschung, N27 Raum 00.008, University Medical Center Hamburg-Eppendorf, Martinistr. 52, 20246 Hamburg, Germany; 6 Max Planck Unit for the Science of Pathogens, 10117 Berlin, Germany; 7 Departments of Health Sciences and Biological Sciences, Faculties of Applied Health Sciences and Mathematics & Science, Brock University, St. Catharines, ON L2S 3A1, Canada

**Keywords:** two-dimensional gel electrophoresis, liquid chromatography, mass spectrometry, splicing, post-translational modification, protein species, protein speciation, proteoform, top-down proteomics, bottom-up proteomics, isoelectric focusing, SILAC, iTRAQ, TMT

## Abstract

Two-dimensional gel electrophoresis (2DE) is an important and well-established technical platform enabling extensive top-down proteomic analysis. However, the long-held but now largely outdated conventional concepts of 2DE have clearly impacted its application to in-depth investigations of proteomes at the level of protein species/proteoforms. It is time to popularize a new concept of 2DE for proteomics. With the development and enrichment of the proteome concept, any given “protein” is now recognized to consist of a series of proteoforms. Thus, it is the proteoform, rather than the canonical protein, that is the basic unit of a proteome, and each proteoform has a specific isoelectric point (p*I*) and relative mass (*M_r_*). Accordingly, using 2DE, each proteoform can routinely be resolved and arrayed according to its different p*I* and *M_r_*. Each detectable spot contains multiple proteoforms derived from the same gene, as well as from different genes. Proteoforms derived from the same gene are distributed into different spots in a 2DE pattern. High-resolution 2DE is thus actually an initial level of separation to address proteome complexity and is effectively a pre-fractionation method prior to analysis using mass spectrometry (MS). Furthermore, stable isotope-labeled 2DE coupled with high-sensitivity liquid chromatography-tandem MS (LC-MS/MS) has tremendous potential for the large-scale detection, identification, and quantification of the proteoforms that constitute proteomes.

## 1. Introduction

After several attempts to improve protein resolution by combining different electrophoretic methods [1,2,3,4,5,6,7], in 1975, O’Farrell published the combination of isoelectric focusing using sodium dodecyl sulfate-polyacrylamide gel electrophoresis (SDS-PAGE) [8]. This combination resulted in a breakthrough in resolution where about 1000 spots could be resolved in one gel. This combination was named two-dimensional gel electrophoresis (2DE). Until quite recently, 2DE has largely been used to resolve and array proteins according to their isoelectric points (p*I*) and relative masses (*M_r_*). The separated proteins, visualized as 2DE “spots” via the use of different staining reagents, thus form a two-dimensional protein map. The difference in spot volumes of matched gel-spots between two different given conditions, such as disease versus normal controls, is used to identify total alterations in protein abundance or changes in the abundance of certain post-translational modifications (PTM) with selective stains; this was primarily based on the conventional concept that 2DE is a high-resolution analytical tool yielding only one or two proteins per spot. Thus, the spot volume represented the combined abundance of each protein species. The difference in spot volumes between matched spots was said to indicate the protein abundance alterations between two different biological situations [9,10]. Excised protein spots of interest could then be subjected to in-gel digestion with trypsin (or other proteases) and identified using analyses coupling peptide mass fingerprint (PMF) or tandem mass spectrometry (MS/MS) and available databases. This has thus been one of the main analytical strategies to address the complexity of proteomes even before the concept of the proteome appeared in 1995 [11]. With continuous improvements to the overall methodology from a number of groups over the last 40 years, 2DE is the current gold standard for the optimal resolution of proteoforms that underlie proteome complexity; 2DE is thus a critical technical platform—indeed, the most well-established approach—for detailed top-down proteomic analyses. Methodological optimization began with improvements of reproducibility and resolution, reaching 10,000 spots in controlled running conditions and with very large gels (30 cm × 40 cm) [12,13]. The development and commercialization of immobilized pH gradient (IPG) strips [14], such as Bio-Rad and GE Healthcare products, significantly improved the ease and reproducibility of the isoelectric focusing (IEF) step, helping to establish the 2DE methodology in many laboratories all over the world. The multi-gel SDS-PAGE system was introduced to significantly improve reproducibility in the second dimension of resolution [15,16]; it was also established that non-linear (NL) pH 3–10 IPG strips were generally superior to the linear gradients and that a constant gel porosity in the second dimension was better than a gradient one with respect to the separation of proteoforms in the analysis of pituitary samples [15,16]. Meanwhile, some studies demonstrated that more spots were resolved with linear gradient IPG strip in the first dimension and there was a much better resolution with a gradient gel in the second dimension in analysis of serum samples [17,18]. 2DE protocols are also being continuously and rigorously refined [19,20,21,22,23] to improve the resolution and subsequent MS-identification, including the use of prefractionation [24] and third electrophoretic separations as a post-fractionation strategy [19], optimization of detergent composition in the solubilization buffer [20,25,26,27,28,29,30,31], pre-extraction of sample handling using automated frozen disruption [21], sampling of tissue with a picosecond-infrared laser (PIRL) technology for getting a better access to the original composition of proteoforms in a tissue [32], a routine “deep imaging” approach to resolve and detect even low-abundance species with 2DE [22], and the newer Coomassie formulation stain/wash protocol and UV-detection as the highest sensitivity protocol for in-gel protein detection, providing detection of intact proteoforms in the low-to-sub-femtomole range (i.e., comparable to the detection of peptides in routine MS analyses) [23]. Indeed, these improved 2DE methods have enabled a newfound respect for the true complexity of proteomes and for the depth of analysis necessary to genuinely understand molecular mechanisms and identify the best possible biomarkers. However, such analyses must go well beyond the still widely held conventional concept that there are only one to two proteins in each 2DE spot in the analysis of the complex proteome [33,34,35]. It is time for the field of proteomics to recognize the critical complementarity of different available approaches, as already emphasized in 2004 [36]. The pros and cons of each method must be fully understood and respected.

**Figure 1:**
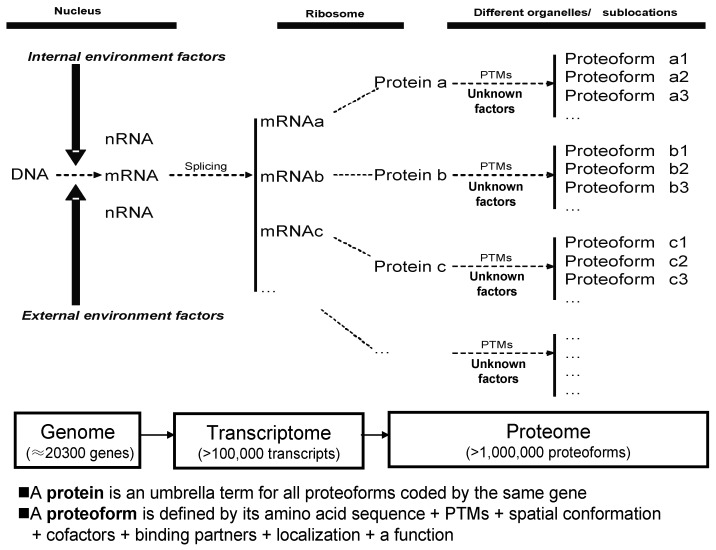
Relationship of proteoform, protein, and proteome. PTM: Post-translational modifications. Modified from Zhan et al. [35], with permission from Hapres publisher open access publication, copyright 2018.

The term protein species [37,38,39], now often also referred to as a proteoform [40], introduced the view that protein species/proteoforms rather than proteins (i.e., the canonical amino acid sequence alone) are the basic units of a proteome. Protein species is a historically grown and chemically defined term [38,39]. Proteoform is a one-word and gene-centric term, which is currently widely accepted and already often used. Despite subtle original differences in the interpretations that led to the use of the terms “protein species” and proteoform, they are now most widely accepted as essentially conceptually identical [34,35,41], or proteoform is nearly identical with protein species. The products of a single gene actually represent a set of proteoforms, each of which has a specific p*I* and *M_r_*, and can thus be resolved using 2DE [34,35]. Here, the term protein species/proteoform is further clearly defined as a primary amino acid sequence + PTMs + spatial conformation + cofactors + binding partners + localization + a clearly defined function (Figure 1). Furthermore, multiple variable transcripts (e.g., splicing variants) correspond to a single gene, and each transcript can be translated into a primary amino acid sequence, each of which may then also consist of multiple proteoforms. Overall, then, proteoforms derived from the same gene can be distributed within different 2DE spots according to variations in p*I* and *M_r_*, and a given spot may be expected to contain multiple proteoforms derived from different genes. A visible given spot might thus consist of many tiny spots barely resolved from each other, and certainly not visually distinguishable with current technology. Furthermore, while there have been different processes used to estimate the size of the human proteome [42,43] with values ranging from at least one million proteoforms in a given cell type [44], up to even ≈1 billion proteoforms [45], it should come as no surprise that each gel spot may contain many proteoforms with the same or very similar p*I* and *M_r_*. While there has been some early evidence consistent with this state of complexity [19,44,46,47,48], a recent study has unequivocally confirmed this phenomenon [33,34], finding that every spot in a 2DE map of a complex native human proteome contains on average fifty to several hundred proteoforms. Many if not all proteins derived from the same gene are distributed within different 2DE spots, and most species in each gel spot are of low-abundance [34]. Some studies also used a cutting 2D gel pixel (10 mm × 6.66 mm × 1 mm) strategy and identified an average of five proteoforms corresponding to each gene in HePG2 cells [49] and an average of four proteoforms corresponding to each gene in glioblastoma cells [50]. These findings, made possible with high sensitivity mass spectrometers, have now completely broken through the conventional concept of only one to two proteins in each 2DE spot in the analysis of complex proteomes [35]. If stable isotope labeling, such as isobaric tags for relative and absolute quantification (iTRAQ), tandem mass tags (TMT), and stable isotope labeling of amino acids in cell culture (SILAC), are incorporated into the 2DE-LC-MS methodology, then stable isotope-labeling 2DE-LC-MS/MS is expected to be an extremely powerful approach for the large-scale detection, identification, and quantification of proteoforms [35]. An alternative approach might be 2DE-LC-MS/MS using label-free quantification; although this would require a much larger number of LC-MS/MS runs, it is devoid of the issues associated with some of the pre-labelling techniques and would be immediately implementable for any sample type.

In summary 2DE-LC-MS/MS provides the most in-depth analysis of proteomes currently available. Further optimization is still possible and reasonably straightforward to achieve. Therefore, innovation in the conception and practice of 2DE, as well as in high-throughput/high-resolution LC-MSMS, will be essential to effectively address the real complexity of proteomes.

## 2. Definition of Spots and Pixels

**Figure 2:**
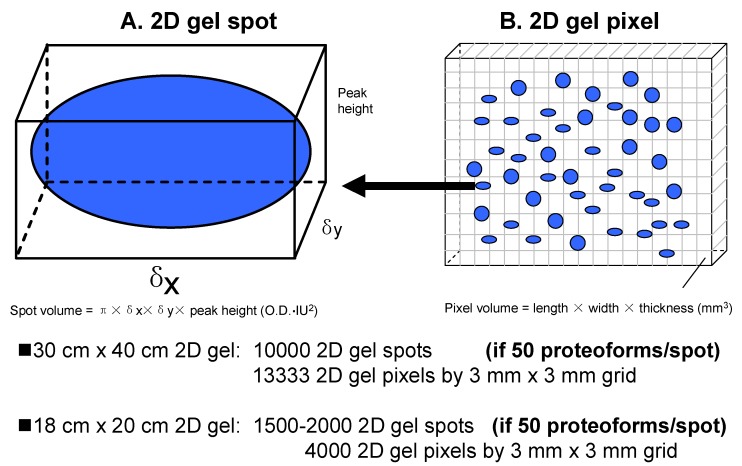
The model of gel spots and gel pixels in a 2DE map. (**A**) The model of a 2D gel spot; (**B**) The model of a 2D gel pixel.

Generally, a 2DE spot is a visible dot in a 2DE map, which is generated by 2DE-resolved proteoforms at a certain location (i.e., grid reference of p*I* and *M_r_*). A spot is most generally visualized using stains, such as Coomassie [33] or silver [51], or using fluorescent stains, such as Sypro Ruby and Flamingo [52,53,54] (Figure 2A). Since a resolving gel has a certain thickness (commonly 1 mm), a given spot has a volume described by a Gaussian distribution, with spot height × πδ_x_δ_y_ (OD·IU^2^) [16]. Spot height is the peak value of the Gaussian spot, and its unit is optical density (OD). δ_x_ is the standard deviation of the Gaussian distribution of the spot in the x-axis direction in image units (IU). δ_y_ is the standard deviation of the Gaussian distribution in the y-axis direction (also in IU). One IU = 100 micrometers (100 μm) = 0.1 mm (0.1 mm). The units of the spot volume are OD × IU^2^. One must note that a specific proteoform will resolve to a single very tiny spot that is resolved from others. However, any proteoforms with very similar p*I* and *M_r_* will migrate to the identical position in the gel. A given visible spot on a 2DE gel can thus be understood as a tight and overlapping group of very tiny spots [35].

Every protein staining method has its own sensitivity (i.e., limit of detection) [23,42,55,56,57]. Spot detection thus correlates directly with the total amount of proteoforms present and the optical density of a spot does not give any information about the amount of any of the single components. In reality, the dynamic abundance range of proteoforms is very large, ≈12 orders of magnitude [58,59]. Considering the inherent complexity of proteomes, detection sensitivity, as well as the theoretical plates of separation for a given gel, it can be expected that spot-free regions of the gel contain rare proteoforms because many low-abundance proteoforms do not reach the detection limit of the protein-staining methods used. Therefore, one might cut the 2DE gel into multiple different pixel-sized pieces using a grid (e.g., 3 mm × 3 mm) for subsequent MS analysis [60] (Figure 2B). A gel pixel has a volume, with length (e.g., 3 mm) × width (e.g., 3 mm) × thickness (commonly 1 mm). Of course, the pixel size should be optimized according to the workload and detection sensitivity of the available 2DE-LC-MS/MS platform. Here, we initially assume a pixel size of 3 mm × 3 mm × 1 mm; it also might be 1 mm × 1 mm × 1 mm, 2 mm × 2 mm × 1 mm, 3 mm × 5 mm × 1 mm, or other sizes. For example, a big pixel size 10 mm × 6.66 mm × 1 mm (a total of 96 sections from a 2D gel 8 cm × 8 cm × 1 mm) was used to analyze HePG2 cells with the achievement of 20,462 proteoforms encoded by 3774 genes [49], and glioblastoma cells with the achievement of 16,012 proteoforms encoded by 4050 genes [50]. A pixel contains all proteoforms with a very similar p*I* and *M_r_* in a given grid area [61,62]. Such a vision of a molecular scanner [60] was not realized until now because even with the available LC-MS techniques, this would be a very complex, time-consuming, and expensive cataloging exercise, but is ultimately necessary if we are aiming to decipher the proteome at the level of proteoforms.

## 3. Relationship between Proteoform, Protein, and Proteome

The apparent completion of the analysis of the human genome [63] has resulted in the identification of about 20,300 genes (although there are caveats to this estimate [64,65,66]), and has driven researchers into the era of transcriptomics and proteomics to study phenotypes and their potential linkage back to the genome. Due to RNA splicing and other factors, many transcripts are often derived from a single gene [67,68,69]; thus, at least 100,000 transcripts are contained in the human transcriptome [44,70]. Many of the newly synthesized proteins, after leaving the ribosome, are then processed, e.g., by proteolytic cleavage of a signal peptide and modified by one or more post-translational modifications (PTMs); at this point there are more than 400 known PTMs [70]. After being modified, the proteoform reaches its specific subcellular location and selectively interacts with surrounding molecules, sometimes engaging in a complex formation, to carry out its specific biological function. This often transient final structural and functional form of a protein is a protein species/proteoform [38,39,40]. Thus, a protein is in reality a set of proteoforms [35]. Each proteoform is the specific and ultimate form of a protein. Proteoforms, but not proteins, are the basic units of any proteome, and are the long-range final functional effectors of a gene (Figure 1). Given sufficient abundance, each proteoform, with its specific p*I* and *M_r_*, can be resolved and detected using 2DE, and identified with coupled MS/MS.

## 4. A 2DE Spot Contains Many Proteoforms Derived from Different Genes

**Figure 3:**
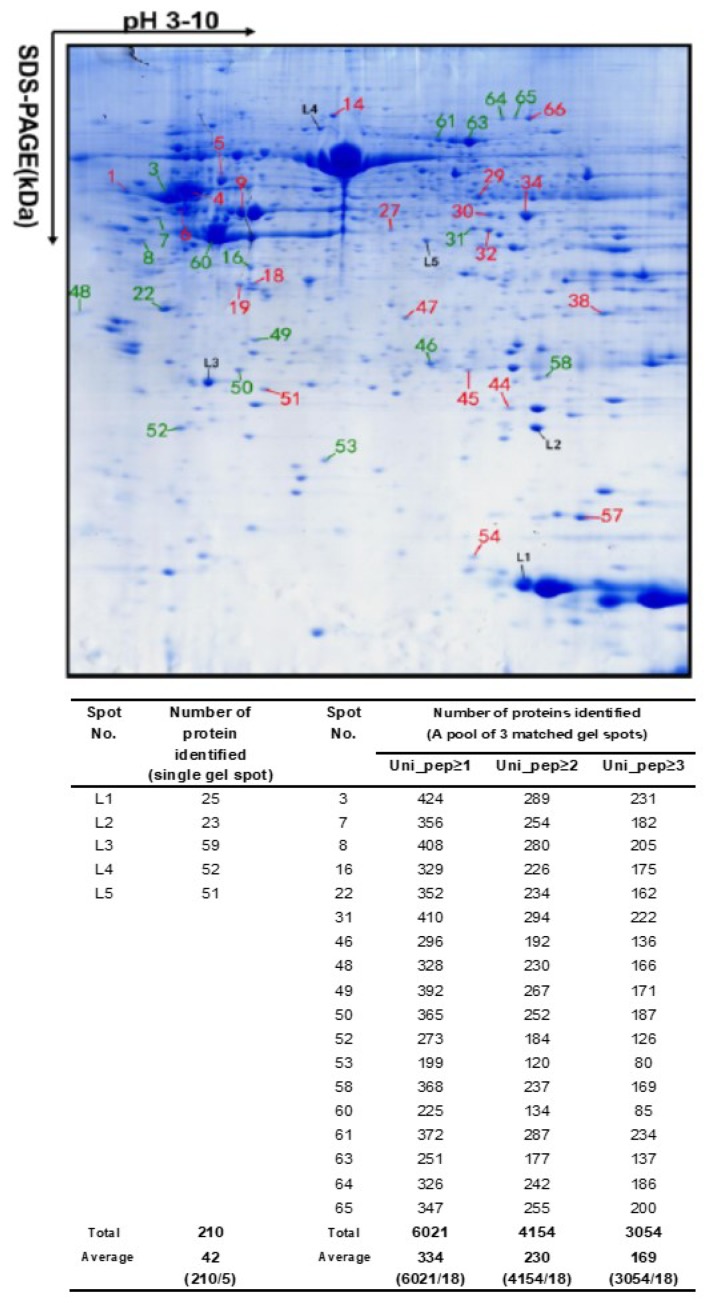
An average of over 50 to several hundred protein species were identified in every two-dimensional gel electrophoresis (2DE) spot of the human glioblastoma proteome. The glioblastoma proteome was resolved using 18 cm pH 3–10 non-linear immobilized pH gradient gel (NL IPG) strips and 12% sodium dodecyl sulfate-polyacrylamide gel electrophoresis (SDS-PAGE) in the second dimension, and stained with Coomassie Blue. Spots L1–L5 came from one gel for tandem mass spectrometry (MS/MS) analysis. Each spot labeled with a red or green number was combined from three matched spots from parallel gels for MS/MS analysis. Modified from Zhan X. et al. [34] with permission from Wiley-VCH.

Many proteoforms with very similar p*I* and *M_r_* exist in proteomes and can comigrate into a 2DE pixel. Different proteoforms in a single 2DE pixel usually have significant abundance differences. With the use of high sensitivity mass spectrometers, these low-abundance proteoforms can be detected, identified, and quantified. A recent study experimentally confirmed this principle, where every 2DE spot contained about 50 proteoforms on average, sometimes several hundred of them at maximum [33,34] (Figure 3). This completely breaks through the conventional concept of assuming only one to two proteins (which in reality are different proteoforms) in each gel spot in a 2DE map, although, in some gel spots of a 2DE map, several proteins had already been found [19,46,48,50,61]. In 2013, Thiede et al. [47] reported that 50% of the spots contained more than one protein (most spots: 2–3 proteins/spot, and only one spot: 22 proteins/spot). In 2015, the Zhan research group first reported that every identified spot contained over fifty proteoforms [33], and then in 2018, confirmed that every 2DE spot analyzed contained over fifty and even up to several hundred proteoforms [34]. Together, these results completely changed the traditional concept of 2DE for proteome analysis, which was addressed in detail in 2018 [35].

## 5. Proteoforms from One Gene are Distributed within Different 2DE Spots

**Figure 4:**
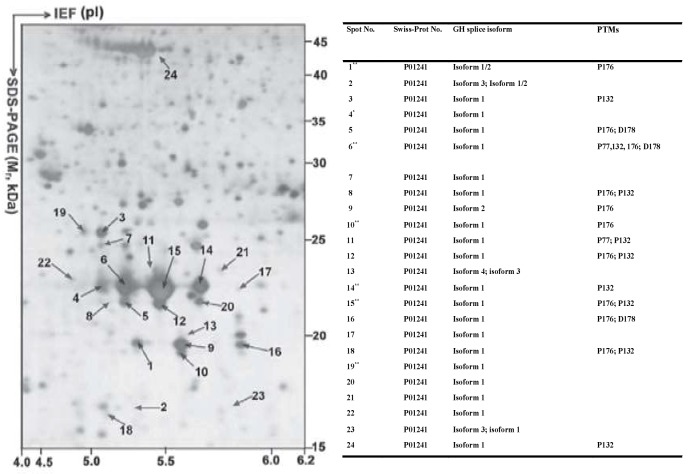
A total of 24 hGH proteoforms are found in the 2DE map of the human pituitary proteome. The human pituitary proteome was resolved as described in Figure 3 and silver stained. IEF: isoelectric focusing. Modified from Zhan X. et al. [71] with permission from Wiley-VCH.

Many proteoforms arising from the same gene, but each with a specific p*I* and *M_r_*, are expected to be resolved into different 2DE spots [71,72,73,74]. For example, (i) 24 human growth hormone (hGH) proteoforms (each GH proteoform identified with more than 3 peptides) derived from the same GH gene are present in 24 different spots in the 2DE map of the normal human pituitary proteome (Figure 4) [71]; (ii) 6 human prolactin (hPRL) proteoforms derived from the same hPRL gene are present in 6 different spots in the pituitary proteome 2DE map [72]; (iii) 59 HSP27 proteoforms derived from the same HSP27 gene are present in 59 different gel spots in the 2DE map of the human myocardium proteome [73]; (iv) 52 HSP70 proteoforms derived from the same HSP70 gene, 24 gamma-enolase-2 proteoforms derived from the same gamma enolase-2 gene, and 17 lactate dehydrogenase 2B proteoforms derived from the lactate dehydrogenase 2B gene are all present in different gel spots in the 2DE maps of the mouse brain proteome [74]; and (v) 28 and 29 proteoforms of lamin A/C and vimentin, respectively, have been identified in a 2DE map of Hela cells [47].

## 6. Most Proteoforms in a 2DE Spot are of Low-Abundance

With an upper estimate of over 1 million proteoforms in a given cell type of the human organism [44], the dynamic range of these proteoforms is estimated to be up to 10^12^ [58,59]. The deep-dive study confirmed that most proteoforms are of low-abundance among the about fifty-to-several hundred protein species found in a single given 2DE spot (Table 1) [34]. The important correlate of this is that that species most easily identified (i.e., having the best sequence coverage) is also the most abundant in any given spot and thus also most likely to be the species changing in abundance when differences in spot density are assessed (i.e., between experimental conditions) [47,74]. Overall, the results thus clearly demonstrate that 2DE in combination with LC-MS/MS is a robust, state-of-the-art, top-down approach to resolve and detect low-abundance proteoforms in complex proteomes, but also to carry out routine comparative proteomic analyses.

## 7. Stable Isotope-Labeled 2DE-LC-MS/MS for the Large-Scale Analysis of Proteoforms

**Figure 5:**
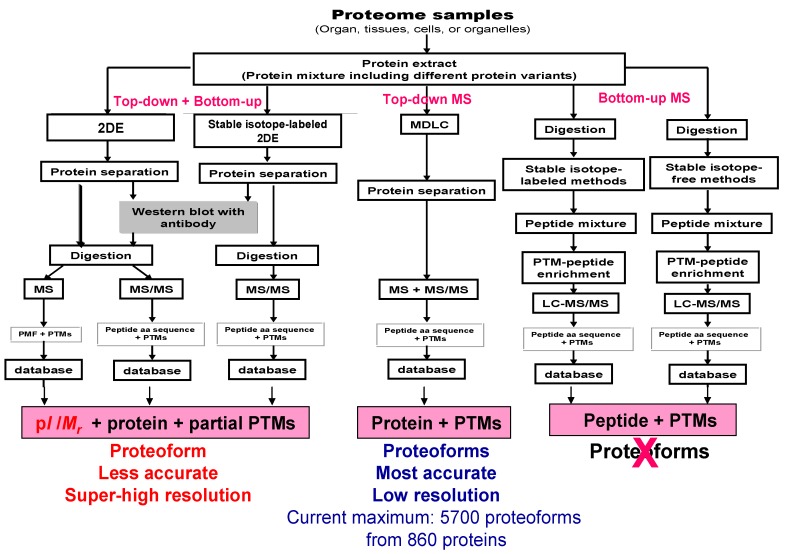
Comparison of methods to study proteoforms. 2DE is a top-down analysis. MS/MS analysis after enzymatic digestion is a “selective” bottom-up analysis. 2DE—two-dimensional gel electrophoresis, MDLC—multiple dimensional liquid chromatography, MS—mass spectrometry, MS/MS—tandem mass spectrometry, PTM—post-translational modification, LC—liquid chromatography, PMF—peptide mass fingerprint. Modified from Zhan X. et al. [70] with permission from Elsevier publisher open access publication, copyright 2018.

Having established that 2DE-LC-MS/MS is the most effective available method to address the sheer complexity of proteomes via the large-scale resolution, detection, and identification of intact proteoforms in the analysis of the complex proteome [34], what are the current limits of the approach without additional refinements? Approximately 10,000 2DE spots can be resolved using a 40 cm tube gel in the first dimension of IEF separation and a 40 cm × 30 cm gel in the second dimension of separation [12], or 13,333 gel pixels can be obtained using a 3 mm × 3 mm grid for such a gel. Even for an 18 cm × 20 cm 2DE gel with an 18 cm pH 3–10 nonlinear (NL) IPG strip, commonly 1500–2000 2DE spots or 4000 gel pixels (3 mm × 3 mm grid) are routinely achievable (Figure 2). Similarly, capitalizing on more recent refinements to a 2DE protocol, including separate resolutions for the soluble and membrane sub-proteomes, high-sensitivity near-IR detection of proteoforms, 3D separations of p*I* extremes and regions of protein hyper-abundance, and deep imaging, a total of 2500–3000 spots were detected using a mini-gel format (i.e., 7 cm IPG strips and 82 mm × 58 mm × 1 mm SDS-PAGE); using a conservative estimate of 50 intact proteoforms per spot [34], this indicates a total proteoform resolution of ≈125,000–150,000 species, which is many times the resolution of any other currently available analytical approach. Estimates of proteoform resolution using large format 2DE thus extend from approximately 500,000 to a million or more proteoforms, values that are unlikely to be achieved by approaches using bottom-up only (which nonetheless do not routinely identify proteoforms) or alternate “in-line MS” top-down analyses that still require front-end separations, deal poorly with hydrophobic proteins, and do not permit any large-scale analysis of proteoforms larger than ≈40 kDa [75,76,77,78,79,80]. The best published detection using capillary zone electrophoresis and in-line top-down MS is 5700 proteoforms from 860 proteins [81,82]. Nonetheless, both the refinements to the 2DE protocol (i.e., 3D separations and deep imaging) in combination with subsequent high-sensitivity bottom-up MS/MS (LC-MS/MS) (i.e., OrbiTrap mass spectrometers) provide for the routine identification of a much larger number of proteoforms at very different levels of abundance (Figure 5). Thus, even considering the important technical advancements contributed by bottom-up approaches over the last decade, 2DE-LC-MS/MS is opening a new horizon that is central to the necessary deep analysis of proteomes at the level of proteoforms.

To ensure the best possible quantitative proteome coverage and the fullest possible sequence and PTM coverage of any given proteoforms, in order to link specific proteoforms to specific biological roles in different physiological or pathological states, further refinements are necessary. The most immediately obvious again capitalize on the complementarity of available methods. The first, quantitative approach would be to quantify each proteoform using stable isotope-labeling of native proteomes before 2DE-LC-MS/MS analysis [35]. For example, SILAC reagents can be used to label all the proteoforms in cultured cells under different conditions, followed by 2DE-LC-MS/MS analysis (Figure 5) [47]. An alternative method, more broadly applicable to the range of sample types encountered in proteomic analyses (i.e., tissues, body fluids), might be stable isotope-labeling of the extracted proteoforms [83,84,85,86], again followed by 2DE-LC-MS/MS analysis (Figure 5 and see below).

One must, however, realize that while SILAC-2DE-LC-MS/MS works well in cell culture [47], it is not applicable to tissue extracts or biological fluids. Alternatively, commercially available isobaric tags (iTRAQ or TMT) [83,84,85,86] or isotope-coded affinity tags (ICAT) can be used to label protein amine groups (i.e., the N-terminus and lysine side-chains) or cysteines, respectively, followed by 2DE-LC-MS/MS analysis [83,84,85,86,87]. However, currently, caveats regarding labeling conditions and the potential for non-quantitative labeling of species must be taken into account; in this regard, such deep proteome analyses may be the most effective test of how such reactive labeling approaches actually quantitatively represent the full dynamic range of native proteomes (i.e., are very low abundance species represented as effectively as high abundance species or is such labeling dominated by the law of mass action?). Isotope-free methods are another choice to quantify proteins with 2DE-LC-MS/MS, including label-free methods [75,88], selected reaction monitoring (SRM) [89], and sequential window acquisition of all theoretical mass spectra (SWATH) [90]. Label-free quantitative mass spectrometry (LFQ-MS) may be a good option with data-dependent acquisition (DDA) [91,92] to compare changes in the abundance of a specific proteoform between two different treatments or physiological conditions; this is perhaps the most straightforward of approaches considering the prior resolution of proteoforms by 2DE. However, for such an approach to be fully effective, new very complex algorithms will be needed considering the multiple dimensions of resolution (i.e., 2DE dimension, LC-MS dimension, and MS/MS dimension). Nevertheless, such an approach would best capitalize on the complementarity of available analytical methods, such as data-independent acquisition (DIA)-based SWATH methods [90].

## 8. Conclusions

Proteoforms are the basic units of the proteome, the critical players defining molecular mechanisms, and the best possible biomarkers; they are thus of central importance across the spectrum of biological, biomedical, agricultural, and environmental research [35,41,44,76,93]. 2DE has undergone significant innovations, both in practice and concept, and its recent further state-of-the-art combination with LC-MS/MS analyses of the highest available sensitivity has firmly established 2DE-LC-MS/MS as the most rigorous approach to deep, quantitative proteome analysis at the proteoform level. The applicability of SILAC-2DE-LC/MS has already been shown in the search for proteoforms changing in abundance during apoptosis [47]. In the future, such routine quantitative analyses will be further complemented using established labelled and label-free approaches. 2DE-based Western blot with a given protein antibody coupled with LC-MS/MS can also effectively reduce the number of 2DE-derived gel samples to be analyzed with LC-MS/MS as it can detect proteoforms of a given protein in a 2DE matrix [33,72,94]. Overall, the beauty and power of this coupled 2DE-LC-MS/MS methodology is its inherent capacity to capitalize on the combination of refined top-down and bottom-up analytical approaches. The capacity for genuine deep proteome analysis at the critical proteoform level is now a definite reality.

## Figures and Tables

**Table 1:** Most of proteoforms in a 2D gel spot have a low abundance or extremely low abundance in the analysis of the human glioblastoma proteome.

Spot No.	Total (n)	The Number of Proteins by emPAI Range				
		>100 (n)	100–10 (n)	10–1 (n)	1–0.1 (n)	0.1–0.01 (n)
3	289	7	3	28	105	146
7	254	2	6	19	105	122
8	280	2	2	22	130	124
16	226	-	8	32	89	97
22	234	1	3	27	122	81
31	294	-	4	36	125	129
46	192	1	4	19	106	62
48	230	-	1	29	132	68
49	267	3	2	29	139	94
50	252	-	1	40	126	85
52	184	1	-	33	99	51
53	120	-	-	12	61	47
58	237	2	4	31	129	71
60	134	2	4	6	60	62
61	287	1	6	28	116	136
63	177	1	2	24	53	97
64	242	-	1	18	93	130
65	255	-	1	19	85	150

Note: The number of proteins in each emPAI range, which is the estimation of the ratio of each protein with at least two unique peptides identified in the analyzed glioblastoma 2DE spot with OrbiTrap Velos MS/MS. Each spot was analyzed with a combined three matched gel spots. Each identified protein had at least two unique peptides identified. The emPAI is the exponentially modified protein abundance index (emPAI), which was calculated for each protein in an analyzed spot to estimate the amount of each protein in a 2DE spot. emPAI is equal to 10^PAI^ minus one. The protein abundance index (PAI) is the number of identified tryptic peptides divided by the number of theoretically observable tryptic peptides. Modified from Zhan X et al. [34] with permission from Wiley-VCH, copyright 2018.

## Abbreviations

2DE	two-dimensional gel electrophoresis
DDA	data-dependent acquisition
DIA	data-independent acquisition
hGH	human growth hormone
hPRL	human prolactin
IEF	isoelectric focusing
IPG	immobilized pH gradient gel
ICAT	isotope-coded affinity tags
iTRAQ	isobaric tags for relative and absolute quantification
IU	image unit
LC	liquid chromatography
*M_r_*	relative molecular mass
MS	mass spectrometry
MS/MS	tandem mass spectrometry
OD	optical density
pI	isoelectric point
PTM	post-translational modification
SDS-PAGE	sodium dodecyl sulfate-polyacrylamide gel electrophoresis
SILAC	stable isotope labeling of amino acids in cell culture
SRM	selected reaction monitoring
SWATH	sequential window acquisition of all theoretical mass spectra
TMT	tandem mass tags
